# The mortality of colorectal cancer in relation to the initial symptom at presentation to primary care and to the duration of symptoms: a cohort study using medical records

**DOI:** 10.1038/sj.bjc.6603439

**Published:** 2006-10-24

**Authors:** S Stapley, T J Peters, D Sharp, W Hamilton

**Affiliations:** 1 CAPER Research Practices, Halford Wing, Dean Clarke House, Exeter, EX1 1PQ, UK; 2 Academic Unit of Primary Health Care, Department of Community Based Medicine, University of Bristol, The Grange, 1 Woodland Road, Bristol, BS8 1AU, UK

**Keywords:** colorectal cancer, primary health care, diagnosis, mortality

## Abstract

The association between the staging of colorectal cancer and mortality is well known. Much less researched is the relationship between the duration of symptoms and outcome, and whether particular initial symptoms carry a different prognosis. We performed a cohort study of 349 patients with primary colorectal cancer in whom all their prediagnostic symptoms and investigation results were known. Survival data for 3–8 years after diagnosis were taken from the cancer registry. Six features were studied: rectal bleeding, abdominal pain, diarrhoea, constipation, weight loss, and anaemia. Two of these were significantly associated with different staging and mortality. Rectal bleeding as an initial symptom was associated with less advanced staging (odds ratio from one Duke's stage to the next 0.50, 95% confidence interval 0.31, 0.79; *P*=0.003) and with reduced mortality (Cox's proportional hazard ratio (HR) 0.56 (0.41, 0.79); *P*=0.001. Mild anaemia, with a haemoglobin of 10.0–12.9 g dl^−1^, was associated with more advanced staging (odds ratio 2.2 (1.2, 4.3); *P*=0.021) and worse mortality (HR 1.5 (0.98, 2.3): *P*=0.064). When corrected for emergency admission, sex, and the site of the tumour, the HR for mild anaemia was 1.7 (1.1, 2.6); *P*=0.015. No relationship was found between the duration of symptoms and staging or mortality.

It is generally assumed that making a diagnosis of colorectal cancer as early as possible is beneficial. Earlier diagnosis can arise from screening or improved recognition of symptomatic cancers. In the UK, approximately three-quarters of colorectal cancers present initially to their general practitioner (GP) with non-emergency symptoms, such as rectal bleeding, abdominal pain, or change in bowel habit (Barrett *et al*, 2006). Even after the proposed introduction of screening in the UK in 2006, it is likely that the majority of patients will still present with symptoms (Hamilton *et al*, 2005).

Patients who attend primary care in the UK with clinical features suggesting possible colorectal cancer can be referred to a rapid investigation facility, ‘the 2-week clinic.’ Referral guidance for these clinics outlines clinical scenarios deemed to carry sufficient risk to warrant rapid investigation. (NICE, 2005) The assumption behind these clinics is that cancers have a symptomatic phase of sufficient duration to offer the possibility of earlier diagnosis, leading to mortality benefits. This assumption can be explored by examination of the relationships between mortality (or staging) and the duration of symptoms. Several such studies have been reported. There is a clear relationship between the staging of the cancer and mortality (Mulcahy and O’Donoghue, 1997; Roncoroni *et al*, 1999; Ponz de Leon *et al*, 2000; Gonzalez-Hermoso *et al*, 2004; Olsson *et al*, 2004). Conversely, most studies have shown no relationship between the duration of symptoms and staging or mortality (Stubbs and Long, 1986; Barillari *et al*, 1989; Kyle *et al*, 1991; Mulcahy and O’Donoghue, 1997; Majumdar *et al*, 1999; Roncoroni *et al*, 1999; Kiran and Glass, 2002; Gonzalez-Hermoso *et al*, 2004; Olsson *et al*, 2004; Bharucha *et al*, 2005; Khattak *et al*, 2006; Rupassara *et al*, 2006). Some have reported an inverse relationship, with a shorter duration of symptoms being associated with both worse staging and worse prognosis (Mulcahy and O’Donoghue, 1997; Olsson *et al*, 2004; Rupassara *et al*, 2006). Once emergency admissions, which generally have a shorter duration of symptoms, are corrected for, this inverse relationship disappears (Mulcahy and O’Donoghue, 1997; Olsson *et al*, 2004; Rupassara *et al*, 2006). Only two previous studies have examined the relationship between the first symptom of the cancer and mortality (Gonzalez-Hermoso *et al*, 2004; Korsgaard *et al*, 2006). However, all these previous studies have either dated the onset of symptoms from the dates given in the doctor's referral letter, or by interviewing patients after the diagnosis had been made. These methods are prone to inaccuracy, with some patients able only to specify the calendar year that their symptoms began, and others giving illogical dates of onset, such as dates after their diagnosis had been made (Olsson *et al*, 2004). Furthermore, only two studies were community based, including all cases from a specific area (Olsson *et al*, 2004; Korsgaard *et al*, 2006). The remainder were hospital-based, raising the possibility that cases referred to that hospital were not typical of the whole colorectal cancer population.

We sought to address these methodological issues by an analysis of a cohort of 349 patients with colorectal cancers occurring in a 5-year period from Exeter, Devon, UK (Hamilton *et al*, 2005). In this cohort, all symptoms recorded in primary care before diagnosis were collected and coded systematically. This methodology eliminates concerns about the accuracy of patient recall, although it does not address symptoms experienced but not reported to primary care.

## MATERIALS AND METHODS

### Participants

We studied subjects from a previously described population-based case–control study (Hamilton *et al*, 2005). In that study, all 358 primary colorectal cancer cases in patients aged 40 years or more from Exeter Primary Care Trust (PCT), diagnosed between 1998 and 2002, were identified from the local cancer registry. This list was augmented by computer searches at all 21 general practices in the PCT, identifying a further three cases. Twelve of the total 361 cases could not be studied as they had either died (five) or left Exeter (seven), and their primary care records were unobtainable. Therefore, 349 cases with full primary care records were studied, 141 (40%) of whom had also died by the time of the initial study, but whose records were retrievable. Emergency presentations were defined as those requiring surgical admission for suspected bowel obstruction or perforation, and who had their cancer diagnosed during the admission, almost always at laparotomy. The entire primary care record for the 2 years preceding diagnosis for all subjects was coded using the International Classification of Primary Care-2, (WONCA, 1998) although for this paper only data from the year before diagnosis were used. The second year before diagnosis was omitted because the rate of reporting of the various clinical features was similar in both cases and controls for that year, so reporting of features in that year by cases would probably be unrelated to their future cancer (Hamilton *et al*, 2005).

Survival data were obtained from the cancer registry, up to the 8 December 2005, so were available for between 3 and 8 years. Deaths are routinely notified to the registry. Almost all cancer-related therapy for patients in Exeter PCT takes place at the Royal Devon and Exeter Hospital: attendances to this hospital are also reported to the cancer registry. The last hospital attendance was taken as the last date in which the patient was known to be alive. In 13 patients who had no hospital attendances recorded, nor had been reported dead, their vital status was ascertained from the Patient and Practitioner Services Agency: one patient's details could not be found there, and her status was obtained by direct enquiry at her general practice.

### Clinical features studied

Ten clinical features had been identified as being independently associated with the diagnosis of colorectal cancer: rectal bleeding, abdominal pain, diarrhoea, constipation, weight loss, abdominal tenderness, anaemia (haemoglobin below 13 g dl^−1^), abnormal rectal examination, a blood sugar level over 10 mm l^−1^, and a positive faecal occult blood testing (Hamilton *et al*, 2005). For this analysis, only the five symptoms and one investigation, anaemia, were included. For simplicity, these are called features from now on, with the index feature being the first one present. Three of the other four features: abdominal tenderness, a rectal mass, and a positive faecal occult blood test, would generally be expected to be identified only in patients who had described a symptom. It was therefore more appropriate to study the symptom itself. The final feature associated with colorectal cancer was a raised blood sugar. Clinically, this finding would be of little practical value in identifying symptomatic colorectal cancer, so it was not studied here. Furthermore, the six selected features mirror those used in previous studies, allowing comparisons to be made.

### Analyses

Two research questions were investigated. The first was that the staging and/or mortality of patients would differ depending on the index feature the patient had experienced in the year before diagnosis. Differences in staging by index feature were analysed using ordinal regression. Initially, this was a univariable analysis, which was followed by adding age and sex and emergency admission to the models. In a small number of patients the Duke's staging was unknown. The cancer registry considered that the commonest cause of absent staging was most likely to be because the patient had disseminated disease, with laparotomy and formal staging not having been undertaken. Therefore, a second analysis was conducted, adding the patients whose staging was unknown to the Dukes’ D group. Mortality by index feature was analysed using Cox's proportional hazards regression model. Following a univariable analysis, multivariable Cox regression added the possible confounders of sex, tumour site (to the right of the splenic flexure, or from the splenic flexure to the rectum), and emergency admission. If multiple features were present at the index consultation, the patient was included under each one.

The second issue was that staging and/or mortality would be related to the duration of symptoms, duration being defined as the interval from presentation of the index feature to diagnosis. This was analysed by dividing the duration into approximate quartiles: 1–30, 31–90, 91–180, and 181–365 days before diagnosis. Cox's proportional hazards regression was used to analyse the relationship between duration of symptoms and mortality. The above analyses were then repeated with age, staging, and emergency admissions added to the models. Again, patients with absent staging were added to the Duke's D group and the analyses repeated.

## RESULTS

The cohort contained 349 patients with colorectal cancer. Details of their clinical features, their staging, and whether they had an emergency presentation are shown in Table 1. Age and sex distributions were similar between elective and emergency presentations: *P*=0.65 (Wilcoxon test) and 0.21 (*χ*^2^), respectively. There was also no relationship between age and staging: *P*=0.35 (median test). This remained so even when the unknown staging cases were added to Duke's D group. Similarly, there was no relationship between sex and staging: *P*=0.91 (*χ*^2^) again without a material difference when the unknown staging cases were added to the Duke's D group. Three hundred and nineteen of the 349 cases had reported one of the features: of these 319, a single feature was noted at the index consultation in 255 (73%), two features in 50 (14%), and three or more in 14 (4%). In the 319 cases with one of the features, the median time this feature was noted was 97 days before diagnosis (interquartile range (IQR) 44, 218).

### Initial symptoms and staging

The Duke's staging in relation to the initial symptoms is shown in Table 2. The ordinal regressions suggested two initial features were associated with different staging. The first was rectal bleeding with an odds ratio of 0.50 (95% confidence interval 0.31, 0.79), *P*=0.003, meaning that rectal bleeding was associated with earlier staging. The second feature was mild anaemia with an odds ratio of 2.2 (1.2, 4.3), *P*=0.021, meaning mild anaemia was associated with later staging. These results were almost unchanged if the patients with unknown staging were added to the Duke's D group, or when age, sex, or emergency admission status were added to the models.

### Initial symptoms and mortality

Of the 349 cases, 207 (59%) had died by the date of this study. The deaths had occurred a median 421 (IQR 102, 841) days after diagnosis. Follow-up data were available in survivors for a median 1592 (1193, 2001) days after diagnosis. There was no relationship between the number of symptoms recorded at the index consultation and mortality (Table 3).

### Staging and mortality in relation to the duration of symptoms

Of the 319 patients with at least one feature of cancer present in the notes, the first feature was recorded between 30 days and 1 day before diagnosis in 61 patients (17% of the cohort), between 31 and 90 days in 92 patients (26%), between 91 and 180 days in 62 patients (18%) and between 181 and 365 days in 104 (30%) (Table 4).

There was no apparent relationship between the duration of symptom in quartiles and staging: *P*=0.27 (*χ*^2^ test, 12 degrees of freedom (d.f.)). This finding was unchanged if those with unknown staging were added to the Duke's D group; *P*=0.67 (*χ*^2^ test, 9 d.f.).

**Figure 1:**
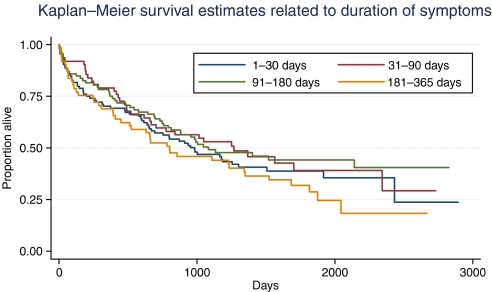
Kaplan–Meier survival estimates in relation to symptom duration.

Mortality in relation to symptom duration is shown in Figure 1. There was no relationship between the duration of symptoms and mortality: *P*=0.47 (Cox proportional hazards). In a multivariable model including symptom duration, there was a relationship between mortality and emergency admission (hazard ratio (HR) 1.9: *P*<0.001), age (HR 1.03 for each year older: *P*<0.001), and staging (HR 1.5 for each increase in stage: *P*<0.001). In this model, left-sided tumours had a survival advantage of marginal significance (HR 0.75: *P*=0.059) but again symptom duration was not associated with mortality (HR 1.0: *P*=0.11).

## DISCUSSION

The main findings from this study are that the mortality from colorectal cancer differs depending upon the nature of the first clinical feature reported to primary care. Patients who experience rectal bleeding have a lower mortality, whereas those who have mild anaemia have a higher mortality. In contrast to these findings, no relationship was found between the duration of symptoms and mortality, even when corrected for possible confounders, such as having an emergency admission.

### Strengths and weaknesses

This is a large community-based study without recall bias, and with no patients lost to follow-up. Even so, some of the subgroups contained relatively small numbers, reducing power for some of the analyses. It is the first study to use contemporary medical records to validate patients’ symptom experiences before colorectal cancer is diagnosed. As such, identifying the interval between symptom reporting and diagnosis is probably more accurate than in previous studies, all of which have relied upon patient recall. There are three caveats, however. Firstly, patients may have experienced symptoms, yet not reported them to the doctor. This may be relatively unimportant in terms of reducing delay in investigation, as current initiatives are based on expediting the diagnostic process once a symptom has been presented, rather than encouraging earlier presentation of symptoms by patients. The second caveat is that patients may have described symptoms to their doctors, which then went unrecorded. We cannot know how often this happened, but it is encouraging that the incidence of rectal bleeding reports in the original study matched previous reports almost exactly (Hamilton *et al*, 2005). The third caveat is that we defined the first symptom occurring in the year before diagnosis as being the index symptom. Some of these first symptoms – abdominal pain in particular – may have been unrelated to the cancer. However, less than one in ten healthy patients report abdominal pain to primary care in any 2-year period, (Hamilton *et al*, 2005) so it is unlikely that more than a few of the 131 (38%) of patients in whom abdominal pain was the first feature noted were in fact misclassified.

### Comparison of findings with previous literature

This study is very similar to previous series in terms of symptoms reported by patients. The staging of the tumours and the proportions with left- or right-sided tumours or with emergency admissions are also similar. The finding of no relationship between the duration of symptoms and staging or mortality – even with our robust methods of identifying the duration – matches previous reports (Stubbs and Long, 1986; Barillari *et al*, 1989; Kyle *et al*, 1991; Mulcahy and O’Donoghue, 1997; Majumdar *et al*, 1999; Roncoroni *et al*, 1999; Kiran and Glass, 2002; Gonzalez-Hermoso *et al*, 2004; Olsson *et al*, 2004; Bharucha *et al*, 2005; Khattak *et al*, 2006; Rupassara *et al*, 2006). It is likely that two effects are cancelling each other out: inherent biological features of the tumour and ease of diagnosis. It is likely that there exists a spectrum of biological aggressiveness of the tumour, containing a subgroup of slow-growing tumours with a long duration of symptoms (Jones *et al*, 2001). These cancers would be expected to have favourable staging and mortality, but their symptoms may be vague or atypical, delaying the diagnosis. In this study, nearly half of all patients had symptoms of their cancer not only reported to their doctor, but deemed worthy of record, at least 90 days before the diagnosis. It is possible that advancing the date of diagnosis by even half of that time would yield a mortality benefit, irrespective of any issue of underlying tumour aggressiveness, perhaps by avoidance of emergency admissions in some patients.

The associations between rectal bleeding as an initial symptom and better mortality, and between mild anaemia and worse mortality are important findings. The only other studies to examine outcomes in relation to the initial symptom reported better staging with rectal bleeding (Gonzalez-Hermoso *et al*, 2004; Korsgaard *et al*, 2006), and worse staging with either abdominal pain or anaemia (though anaemia was described in that paper as a symptom, rather than being confirmed by estimation of the haemoglobin, as in the study reported here) (Gonzalez-Hermoso *et al*, 2004). The mortality in relation to these symptoms was not reported in either previous paper, though it would be expected to mirror the staging differences (Gonzalez-Hermoso *et al*, 2004; Korsgaard *et al*, 2006).

Why is rectal bleeding as an initial symptom linked with a good prognosis and anaemia with a poor prognosis? The associations remained present even once the recognised confounders of emergency admission, sex, and the site of the tumour were added to the model, so these variables cannot explain the results. There are two likely explanations. The first possible explanation is that anaemia arises from colorectal cancers as a result of persistent, occult, bleeding into the gastrointestinal tract, so the bleeding (and the tumour) will have been present for some time, perhaps months. In contrast, overt rectal bleeding may occur early in the development of the tumour. A second possibility is that doctors are more alert to the possibility of cancer with rectal bleeding and choose to investigate (du Toit *et al*, 2006), even though current guidance suggests 6 weeks of isolated bleeding is required to warrant urgent investigation (NICE, 2005). Although *severe* anaemia is recognised as a feature of possible colorectal cancer, with a risk of approximately 7% (Logan *et al*, 2002; NICE, 2005), it is still under-investigated in around half of primary care patients. Mild anaemia, with haemoglobin values in the range 10.0–12.9 g dl^−1^, is common in the elderly population, but carries a risk of colorectal cancer of around 1% (Hamilton *et al*, 2005). Many GPs would not even consider the possibility of cancer with such mild anaemia – and others may consider investigation of the gastrointestinal tract inappropriate when the risk is relatively low. Nonetheless, it is likely that our finding of increased mortality from colorectal cancer when anaemia is the initial feature will be perpetuated unless doctors change their investigatory behaviour. Testing the stool of such patients for occult blood would be good clinical practice.

This study also suggests that for most patients with a colorectal cancer there is a sufficient interval after symptoms are reported to the doctor for a worthwhile improvement in the timeliness of diagnosis to be possible. Such an improvement would require doctors to identify that their patient may have cancer, and to request appropriate investigation. This may be particularly pertinent when the patient has mild anaemia. Even though the duration of symptoms is unrelated to the outcome of the cancer, it is impossible to construct an argument in favour of delayed diagnosis.

## Figures and Tables

**Table 1:** Details of the age, sex, emergency presentation, and clinical features of the cohort

		**Number (% of cohort)**	**Median (IQR) age**	**Number (%) male**	**Median (IQR) days before diagnosis**
Entire cohort		349 (100)	73 (65,81)	177 (51)	
Emergency presentations		62 (18)	75 (65,84)	27 (44)	
Duke's staging	A	48	72 (64,80)	25 (52)	
	B	130	75 (66,82)	62 (48)	
	C	100	73 (65,80)	52 (52)	
	D	46	70 (64,78)	23 (50)	
	Unknown	25	77 (71,83)	15 (60)	
					
First symptom noted	Rectal bleeding	97 (28)	73 (64,81)	56 (58)	64 (29, 133)
	Abdominal pain	82 (24)	70 (61,79)	42 (51)	133 (49, 221)
	Diarrhoea	76 (22)	70 (61,78)	35 (46)	98 (38, 217)
	Constipation	38 (11)	75 (67,81)	22 (58)	126 (59, 222)
	Loss of weight	26 (7)	75 (65,82)	8 (31)	55 (31, 170)
	Mild anaemia	37 (11)	74 (67,80)	20 (54)	133 (81, 305)
	Severe anaemia	43 (12)	79 (75,85)	18 (42)	100 (36, 218)
					
Symptoms noted at any time before diagnosis	Rectal bleeding	136 (39)	73 (65,81)	77 (57)	65 (29, 129)
	Abdominal pain	131 (38)	71 (62,79)	60 (46)	85 (36, 194)
	Diarrhoea	113 (32)	73 (64,79)	51 (45)	78 (33, 181)
	Constipation	80 (23)	73 (63,81)	38 (48)	70 (16, 144)
	Loss of weight	80 (23)	72 (64,80)	34 (43)	47 (20, 104)
	Mild anaemia^a^	80 (23)	73 (67,81)	40 (50)	93 (42, 200)
	Severe anaemia^a^	73 (21)	79 (73,84)	30 (41)	85 (23,168)

IQR=interquartile range.

a Twenty-three of these patients had both mild and severe anaemia at some point before diagnosis. Mild anaemia defined as a haemoglobin 10.0–12.9 g dl^−1^, severe as=<9.9 g dl^−1^.

**Table 2:** Duke's staging in relation to the first symptom

	**Number (%) with each Duke's staging**					
**First symptom**	**A**	**B**	**C**	**D**	**Not known**	*P*-value*
Rectal bleeding	8 (8)	24 (25)	31 (32)	27 (28)	7 (7)	0.003
Abdominal pain	6 (7)	7 (9)	35 (43)	23 (28)	11 (13)	0.59
Diarrhoea	4 (5)	7 (9)	32 (42)	22 (29)	11 (14)	0.50
Constipation	2 (5)	5 (13)	17 (45)	9 (24)	5 (13)	0.64
Loss of weight	0	2 (8)	14 (54)	6 (23)	4 (15)	0.98
Mild anaemia	3 (8)	3 (8)	11 (30)	10 (27)	10 (27)	0.021
Severe anaemia	4 (9)	3 (7)	18 (42)	14 (33)	4 (9)	0.73

* By ordinal regression.

**Table 3:** Mortality in relation to the first symptom

	**Univariable**		**Multivariable^a^**	
**First symptom**	**Proportional hazard (CI)**	***P*-value**	**Proportional hazard (CI)**	***P*-value**
Rectal bleeding	0.56 (0.41, 0.79)	0.001	0.57 (0.41, 0.81)	0.001
Abdominal pain	1.3 (0.93, 1.7)	0.14	1.3 (0.95, 1.8)	0.096
Diarrhoea	0.93 (0.67, 1.3)	0.70	1.1 (0.77, 1.5)	0.63
Constipation	1.3 (0.84, 1.9)	0.27	1.1 (0.79, 1.8)	0.60
Loss of weight	1.5 (0.94, 2.4)	0.092	1.5 (0.91, 2.3)	0.11
Mild anaemia	1.5 (0.98, 2.3)	0.064	1.8 (1.1, 2.7)	0.010
Severe anaemia	0.98 (0.65, 1.5)	0.93	0.79 (0.51, 1.2)	0.30

CI, confidence interval.

a Other variables in model: age, emergency admission status, sex, and site of tumour.

**Table 4:** Duke's staging in relation to the duration of symptoms

		**Number (%) with each Duke's staging**				
**Date of first symptom related to date of diagnosis**	**Number**	**A**	**B**	**C**	**D**	**Not known**
1–30 days	61	8 (13)	23 (38)	19 (31)	6 (10)	5 (8)
31–90 days	92	13 (14)	30 (33)	29 (32)	13 (14)	7 (8)
91–180 days	62	5 (8)	30 (48)	18 (29)	9 (15)	0
181–365 days	104	17 (16)	41 (39)	25 (24)	9 (9)	12 (12)
						
Total	319	43 (13)	124 (39)	91 (29)	37 (12)	24 (8)
